# Genetic studies of extra‐early provitamin‐A maize inbred lines and their hybrids in multiple environments

**DOI:** 10.1002/csc2.20071

**Published:** 2020-05-05

**Authors:** B. Badu‐Apraku, M.A.B. Fakorede, A.O. Talabi, M. Oyekunle, M. Aderounmu, A.F. Lum, P.F. Ribeiro, G.B. Adu, J.O. Toyinbo

**Affiliations:** ^1^ International Institute of Tropical Agriculture P.M.B. 5320 Ibadan Nigeria; ^2^ Department of Plant Science Institute for Agricultural Research Samaru Ahmadu Bello University Zaria Nigeria; ^3^ Department of Crop Production and Protection Obafemi Awolowo University Ile‐Ife Nigeria; ^4^ C/o Bells University of Technology Nigeria; ^5^ CSIR– Crops Research Institute Cereals Division Fumesua Kumasi Ghana; ^6^ CSIR– Savanna Agricultural Research Institute Tamale Ghana

## Abstract

Vitamin A deficiency, drought, low soil nitrogen (low‐N), and *Striga hermonthica* parasitism of maize (*Zea mays* L.) cause malnutrition and food insecurity in sub‐Saharan Africa. The objectives of this study were to determine combining abilities of extra‐early provitamin A (PVA) lines, classify them into heterotic groups (HGs), identify testers, and determine yield stability of hybrids under contrasting environments in two trials. In Trial 1, 190 F_1_ hybrids plus six checks were tested under *Striga‐*infested, drought, and stress‐free environments in Nigeria from 2015–2017. In Trial 2, 35 extra‐early yellow hybrids were evaluated under low‐N, *Striga*‐infested, and stress‐free environments in 2018. TZEEIOR 202 and TZEEIOR 205 had PVA concentrations of 23.98 and 22.56 μg g^−1^. TZEEIOR 197 × TZEEIOR 205 (20.1 μg g^−1^) and TZEEIOR 202 × TZEEIOR 205 (22.7 μg g^−1^) contained about double the PVA level of the commercial check, TZEEI 58 × TZEE‐Y Pop STR C5 (11.4 μg g^−1^). Both general (GCA) and specific (SCA) combining ability variances were significant for most agronomic traits, although GCA was larger than SCA effects, indicating GCA effects primarily controlled the inheritance of those traits. TZEEIOR 97 and TZEEIOR 197 were identified as inbred testers. TZEEIOR 197 × TZEEIOR 205 was identified as a single‐cross tester and the most stable and highest‐yielding hybrid across environments. TZEEIOR 202 and TZEEIOR 205 should be invaluable resources for breeding for high PVA. Provitamin A level was independent of hybrid yield potential, indicating that selection of superior hybrids with elevated PVA levels should be feasible.

**Abbreviations**
ASI: anthesis‐silking interval
ATC: average‐tester coordinate axis
DA: days to anthesis
DAP: days after planting
DS: days to silking
E: environment
EASP: ear aspect
EHT: ear height
EPP: ears per plant
EROT: ear rot
ESP1: emerged *Striga* plants at 8 WAP
ESP2: emerged *Striga* plants at 10 WAP
G: genome
GCA: general combining ability
GEI: G × E interaction
GGE: genotype main effect plus genotype × environment interaction
HGCAMT: Heterotic Grouping based on GCA of multiple traits
HGs: heterotic groups
HPVA: PVA levels of the hybrids
HUSK: husk cover
IITA: International Institute of Tropical Agriculture
MP: mid‐parent pro‐vitamin A
PASP: plant aspect
PHT: plant height
PVA: provitamin A
RL: root lodging
RMHT: Regional Maize Hybrid Trial
SCA: specific combining ability
SDR1: *Striga* damage syndrome ratings at 8 WAP
SDR2: *Striga* damage syndrome ratings at 10 WAP
SL: stalk lodging
SSA: sub‐Saharan Africa
STGR: stay green characteristic
STMA: stress tolerant maize for Africa
VA: vitamin A
VAD: vitamin A deficiency
VAD: vitamin A deficiency
WAP: weeks after planting
WCA: West and Central Africa

## 1 INTRODUCTION

Malnourishment occurs among millions of people worldwide with the larger proportion being in developing countries, especially in Asia and Africa, sub‐Saharan Africa (SSA) in particular (Jauhar, 2006). According to Swaminathan (2012), in some “hunger hot spots” of the world where agriculture is the backbone of survival, as in SSA and South Asia, mainstreaming nutrition in agriculture programs is the most effective and low‐cost method of eliminating malnutrition. During the past two decades, tremendous efforts have been made to improve the nutritional status of crops consumed in SSA, but the region still has the largest number of malnourished people in the world. For instance, in West and Central Africa (WCA), a large proportion of the population has limited access to nutritionally balanced food to support a healthy life (Badu‐Apraku & Fakorede, 2017). Maternal and childhood malnutrition results in underweight individuals, causing millions of deaths in the sub‐region. The World Health Report of 2002 ranked malnutrition first among the top globally preventable health risks, with the dreaded HIV/AIDS ranking fourth, an indication that greater attention should be focused on improving nutrition to minimize the effect of preventable diseases. Cereal‐based diets on which most Africans subsist, have low levels of vitamin A (VA). According to West (2002), about 33 million preschool‐age children suffer from suboptimal VA which has contributed to their vulnerability to several major diseases, including river blindness (onchocerciasis), anemia, diarrhea, measles, malaria, and respiratory infections (Villamor & Fawzi, 2000). Menkir, Gedil, Tanumihardjo, Adepoju, and Bossey (2014) reported that in SSA, vitamin A deficiency (VAD) affects more than 45 million children 5‐yr old or less. Furthermore, VAD impairs the functionality of the immune system, increases susceptibility to diseases, increases the chances of death from severe illnesses, and causes night or complete blindness (Sommer, 2008).

Maize (*Zea mays* L.) is the most important staple food crop in SSA. It is the most widely consumed cereal in WCA, where it is eaten as green maize, processed grain, popcorn, and sweet corn. Quality improvement (bio‐fortification) of this crop, therefore, has a crucial nutritional role in solving some of the problems of malnutrition in Africa. Kernels of some types of maize, especially yellow and orange maize, contain pro‐vitamin A (PVA) in the form of carotenoids in the endosperm, for which there is a high level of genetic variation and which makes it possible to increase accumulation of the vitamin through plant breeding. In a study involving 39 maize inbred lines, Blessin, Brecher, and Dimler (1963) obtained 0.9–4.1 μg g^−1^ of carotenes, and 18.6–48.0 μg g^−1^ of xanthophylls. Ortiz‐Monasterio et al. (2007) reported a variation of 0.24–8.80 μg g^−1^ in total PVA and a range of 5–30% in the proportion of PVA to total carotenoids among 1000 tropical maize genotypes obtained from the International Center for Maize and Wheat Improvement (CIMMYT) in Mexico. Therefore, increasing the level of PVA in maize through breeding is a feasible approach for alleviating malnutrition related to its deficiency. A study was conducted in Zambia by Palmer et al. (2016) to investigate the effect of PVA maize consumption on dark adaptation, an early functional indicator of VAD. Results revealed that children with deficient or marginal VA status showed increased pupillary responsiveness following consumption of PVA maize, thus providing evidence of the functional health benefits of consuming PVA maize.

In addition to VAD, parasitism by *Striga hermonthica*, low soil nitrogen (low‐N), and drought are among other constraints in maize production in SSA. *Striga* parasitism can lead to complete crop failure when the infestation is very severe (Kroschel, 1999). On the other hand, drought could reduce maize yield by up to 90% when it coincides with flowering (anthesis and silking) and/or grain‐filling periods (NeSmith & Ritchie, 1992). Furthermore, the savanna soils, where maize potential could be easily maximized, are low or completely deficient in certain nutrients, such as nitrogen, phosphorus, potassium, several micronutrients, as well as inorganic matter. In SSA, low‐N stress reduces maize grain yield by 10–50% yr^−1^ (Logrono & Lothrop, 1997). Genetic enhancement of maize is the most economic, affordable, and sustainable option for mitigating the adverse effects of *S. hermonthica* parasitism, drought, and low‐N in SSA (Badu‐Apraku et al., 2015a, 2015b, 2015c). The development and commercialization of multiple‐stress‐tolerant maize with high levels of PVA content is urgently required to mitigate VA malnutrition and food insecurity in SSA. Substantial progress has been made in increasing the PVA in maize through conventional breeding. Badu‐Apraku and Fakorede (2017) indicated that the identification of adapted orange maize inbred lines from diverse genetic backgrounds and with varying carotenoid concentrations is critical to facilitating the development of superior PVA hybrids and establishing a successful PVA hybrid program. Suwarno, Pixley, Palacios‐Rojas, Kaeppler, and Babu (2014) demonstrated the effectiveness of grouping PVA lines based on maximum molecular‐marker‐based genetic distance between the lines to achieve heterosis. However, little or no information is available on the development and commercialization of multiple‐stress‐tolerant PVA maize hybrids.

Cultivation of hybrid maize in SSA has occupied center stage in the past decade, and emerging seed companies have relied on existing outstanding germplasm in the public domain for commercialization. However, a successful hybrid development program is a function of the heterotic patterns of the parental lines used in the development of the hybrids and their ability to combine well with most other inbred lines, or specific lines to develop productive and superior hybrids (Fan et al., 2009). Thus, heterotic groups (HGs) are formed among sets of developed inbred lines, such that those with genetic similarity are placed in the same group, whereas those which are genetically dissimilar are categorized in opposite groups (Badu‐Apraku et al., 2015a, 2015b; Fan et al., 2009). This increases the chances of developing outstanding hybrids for commercialization, since crosses are made only among inbred lines from opposite HGs.

Information on inter‐trait relationships guides a breeder on the choice of traits to consider for improving the performance of a primary trait, such as grain yield, under diverse environmental conditions (Talabi, Badu‐Apraku, & Fakorede, 2017). Breeders routinely investigate how grain yield and secondary traits of maize interact, especially when new sets of genetic materials are developed, in order to ascertain that the existing interrelationships among the traits have not been altered by the genetic constitution of the newly developed materials or by climate change. Several researchers have documented inter‐trait relationships in maize. Number of ears per plant (EPP), anthesis‐silking interval (ASI), and stay‐green characteristic (STGR) were identified by Bänziger, Edmeades, Beck, and Bellon (2000) as the most reliable secondary traits for improvement of grain yield under drought‐stress and low N conditions. Badu‐Apraku et al. (2011a) also identified EPP and ASI, along with plant aspect (PASP) and ear aspect (EASP) as the secondary traits for yield improvement under both drought and low N stresses. They also found days to silking (DS), days to anthesis (DA), plant height (PHT), and STGR as indirect selection criteria for grain yield under low‐N environments (Badu‐Apraku et al., 2011a).

Since 2007, several inbred lines with varying levels of PVA and reactions to stresses are being developed in the International Institute of Tropical Agriculture (IITA) maize improvement program. However, there is a dearth of information on the heterotic patterns of the extra‐early PVA inbred lines and on the performance and inter‐trait relationships of derived hybrids in multiple environments. In addition, only few PVA hybrids in the extra‐early maturity group have been developed and released for commercialization in the sub‐region. The studies reported here were therefore conducted to (i) determine the general (GCA) and specific (SCA) combining ability effects for grain yield and several other agronomic traits of extra‐early PVA inbred lines, (ii) assign the inbred lines to appropriate heterotic groups, (iii) identify inbred lines and single‐cross hybrids for use as testers for producing high‐yielding single‐cross and three‐way hybrids, (iv) evaluate grain yield performance and stability of the hybrid combinations of selected inbred lines in drought‐affected, *Striga*‐infested, low‐N, and optimal growing environments, and (v) examine inter‐trait relationships among the PVA hybrids.

## 2 MATERIALS AND METHODS

### 2.1 Development of genetic material

In an effort to develop stress (drought, low‐N, and *Striga*) tolerant and/or resistant, high PVA, extra‐early maturing cultivars for SSA, the *Striga*‐resistant, extra‐early cultivar (42–49 d to flowering), 2004TZEE‐Y STR C_4_ was crossed in 2007 to (Syn–Y‐STR‐34–1‐1–1‐1–2‐1‐B‐B‐B‐B‐B/NC354/SYN‐Y‐STR‐34–1‐1–1; OR1), a source of high PVA, from the IITA Maize Improvement Program in an effort to transfer the genes for high β‐carotene into the cultivar. The F_1_ was backcrossed to the extra‐early cultivar and kernels of the BC_1_F_1_ with deep orange color were selected and advanced to F_2_ and F_3_ stages through selfing. At the F_3_ stage, lines with intense orange color were selected and recombined to obtain the extra‐early PVA cultivar 2009 TZEE‐OR1 STR from which a new set of extra‐early inbred lines were extracted starting in 2011. By 2014, a total of 224 S_6_ inbred lines, selected for deep orange color, had been developed from the variety. This set of PVA inbred lines were assessed for tolerance to induced drought at Ikenne, Nigeria, in the 2014–2015 dry season. Thereafter, the PVA inbred lines were advanced to the S_7_–S_8_ stages from which the kernels were sampled and subjected to chemical analyses at the Food and Nutrition Laboratory of IITA‐Ibadan for the determination of their PVA contents. Results of the chemical analyses were used as the basis for selecting the PVA inbred lines evaluated in the genetic studies reported here.

### 2.2 Trials conducted under contrasting environments

Two trials (Trial 1 and Trial 2) were conducted in the present study. In Trial 1, 20 extra‐early S_7_ PVA inbred lines selected for moderate to high levels of β‐carotene content (Table 1) were inter‐mated in the IITA‐Ibadan breeding nursery in 2015 according to the diallel mating scheme (Sprague & Tatum, 1942), and 190 F_1_ hybrids were obtained. The PVA hybrids and six yellow hybrid checks were used for combining ability studies in *Striga*‐infested (Experiment 1), drought (Experiment 2), and optimal growing environments (Experiment 3) from 2015–2017. Trial 2 was comprised of 34 extra‐early‐maturity genotypes, including PVA and non‐PVA hybrids which were selected from a number of preliminary maize hybrid trials. The 34 hybrids and a commercial check were evaluated in the Stress Tolerant Maize for Africa (STMA) Regional Maize Hybrid Trial (hereafter referred to as Regional Maize Hybrid Trial or RMHT) in *Striga*‐infested, low‐N, and optimal growing environments in Nigeria, in 2018.

**Table 1:** Reactions of 20 provitamin A (PVA) maize inbred lines to *S. hermonthica* and drought, and the PVA contents of the inbreds and some selected hybrids derived from them, along with a commercial PVA check variety

				PVA content		PVA content
Serial no.	Inbred	Reaction to *Striga* a	Reaction to drought a	μg g^−1^	Hybrids	μg g^−1^
1	TZEEIOR 22	T a	Ss	9.28	TZEEIOR 26 × TZEEIOR 97	9.54
2	TZEEIOR 24	T	Ss	9.58	TZEEIOR 26 × TZEEIOR 142	8.85
3	TZEEIOR 26	Ss	Ss	9.74	TZEEIOR 26 × TZEEIOR 197	7.73
4	TZEEIOR 27	T	Ss	7.88	TZEEIOR 27 × TZEEIOR 251	7.90
5	TZEEIOR 28	T	T	11.20	TZEEIOR 30 × TZEEIOR 209	9.44
6	TZEEIOR 30	T	T	10.19	TZEEIOR 30 × TZEEIOR 234	7.65
7	TZEEIOR 41	T	T	11.57	TZEEIOR 41 × TZEEIOR 97	11.29
8	TZEEIOR 45	T	T	9.19	TZEEIOR 109 × TZEEIOR 197	9.48
9	TZEEIOR 97	T	Ss	10.44	TZEEIOR 109 × TZEEIOR 250	9.24
10	TZEEIOR 109	T	Ss	10.24	TZEEIOR 142 × TZEEIOR 250	11.00
11	TZEEIOR 140	T	Ss	10.32	TZEEIOR 197 × TZEEIOR 205	20.1
12	TZEEIOR 142	T	S	9.86	TZEEIOR 197 × TZEEIOR 251	7.94
13	TZEEIOR 197	T	S	8.45	TZEEIOR 202 × TZEEIOR 205	22.7
14	TZEEIOR 202	T	T	23.98	TZEEI 79 × TZEEI 58	2.70
15	TZEEIOR 205	T	T	22.58	TZEE‐Y Pop STR C5 × TZEEI 58 (Check)	11.41
16	TZEEIOR 209	T	T	9.94		
17	TZEEIOR 233	T	S	9.00		
18	TZEEIOR 234	T	S	8.33		
19	TZEEIOR 250	S	T	8.39		
20	TZEEIOR 251	T	T	7.94		

a T, Tolerant and/or Resistant; Ss, Susceptible.

#### 2.2.1 Trial 1: evaluation of PVA hybrids in drought, *Striga*‐infested, and optimal growing environments

This trial consisted of three independent experiments conducted under different management conditions. Experiment 1 involved evaluation of the 190 PVA hybrids plus six extra‐early hybrid checks (42–49 d to flowering) at Mokwa (9°18 N, 5°4 E, 457 m above sea level, 1100‐mm mean annual rainfall) under artificial *Striga* infestation during the 2016 and 2017 growing seasons. Residual *Striga* seeds were eliminated by inducing their suicidal germination through the injection of ethylene gas into the soils 2 wk prior to manual *Striga* infestation. The artificial *Striga* infestation followed the procedure proposed by Kim (1991). Fertilizer application was delayed until about 25 d after planting (DAP) when 30 kg ha^−1^ was applied as N/P/K 15:15:15. Delay in fertilizer application and the low rate of fertilizer were aimed at subjecting the maize plants to a required stress level to trigger the production of strigolactones, the hormones responsible for the stimulation of germination of *Striga* seeds. The *Striga* plants that emerge, being parasitic, grow in the maize field for as long as the host plant (maize) is growing and supplying the required nutrients for the growth and development of *Striga* plants.

In Experiment 2, the 196 hybrids were planted during the dry seasons of 2015–2016, 2016–2017, and 2017–2018 under managed drought at Ikenne (6°53′ N, 30°42′ E, 60 m above sea level, 1200‐mm mean annual rainfall). The soils at Ikenne are characterized as Alfisols (Soil Survey Staff, 2007) which are fairly flat, uniform, and typically have high water‐retention capacity. The experiments were established during the dry seasons of each year, starting from November to March of the following year. Water was provided to the trials through a sprinkler irrigation system which made about 17 mm of water available to each plant every week. Drought was imposed in the trials at about 25 DAP, during which water supply was discontinued. As such, growth and development of plants until harvest were dependent on the moisture stored in the soil. Basal fertilizer was applied as 60 kg each of K, P, and N at sowing. Topdressing was performed by applying 30 kg of N ha^−1^ at 3 wk after planting (WAP).

Experiment 3 involved evaluation of the 196 hybrids in optimal growing environments at Mokwa in 2016, Ikenne in 2016 and 2017, and Bagauda (lat 12°00′ N, long 8°22′ E, 580 m above sea level, 800‐mm mean annual rainfall) in 2017. In the optimal environments, there was adequate supply of water and nitrogen, and the plots were *Striga*‐free. About 60 kg each of N, P, and K ha^−1^ was applied as basal fertilizer at 2 WAP, with additional 30 kg N ha^−1^ top‐dressed 4 WAP.

Each of the three experiments was conducted using a 14 by 14 lattice design, with two replications. In the three experiments, 3‐m‐long single‐row plots were used, with inter‐ and intra‐row spacing of 0.75 and 0.40 m, respectively. Three seeds were sown per hill and emerged seedlings were thinned to two per hill at about 2 WAP to attain the target population density of approximately 66,667 plants ha^−1^. Atrazine (Primextra) was applied for pre‐emergence weed control in all the fields, whereas Paraquat (Gramoxone) served as a postemergence herbicide in drought and optimal fields. Following the emergence of maize plants, S*triga* fields were kept weed‐free through hand pulling.

#### 2.2.2 Trial 2: evaluation of yellow and PVA hybrids in the RMHT under *Striga*‐infestation, Low‐N, and optimal growing environments

Entries of Trial 2 were evaluated in *Striga*‐infested environment in Mokwa during the 2018 growing season. In addition, the trial was conducted in low and high‐N environments at Ile‐Ife and Mokwa, and in optimal environments in Ikenne, Mokwa, Zaria (11°11′ N, 7°38′ E, 640 m) above sea level, 1200‐mm mean annual rainfall), and Bagauda in 2018. The experimental design used was a five by seven lattice with three replications. Two‐row plots were employed, each measuring 4‐m long, with inter‐row spacing of 0.75 m and within‐row spacing of 0.40 m. Other experimental procedures used in the Trial were as given for Trial 1.

The soil of the field used for the low‐N experiment in Mokwa was characterized as Luvisol, and that of Ile‐Ife was characterized as fine‐loamy, isohyperthemic Plinthustalf (Soil Survey Staff, 2007). The fields had been previously depleted of N by continuous planting of maize crop without application of N fertilizer, in addition to the removal of plant residue after each harvest, for a period of 2 yr. Prior to planting, soil samples were collected for determination of N level by the Kjeldahl digestion and colorimetric method using Technicon AAII Autoanalyser (Bremner & Mulvaney, 1982). The soils had very low residual N, varying from 0.21–0.53% N. Based on the soil test results, urea, triple super phosphate, and muriate of potash were used to formulate fertilizer which was applied 2 WAP at the rate of 15, 60, and 60 kg ha^−1^ each of N, P, and K to the low‐N experiments, whereas 45, 60, and 60 kg ha^−1^ of N, P, and K were applied to high‐N experiments. The N‐treatment fields were top‐dressed at 4 WAP with the amount of urea required to increase the total available N to 30 and 90 kg ha^−1^ for low‐N and high‐N experiments, respectively. Herbicides, followed by manual weeding as needed, were employed for weed control in the trial. Evaluation of the hybrids in the RMHT under optimal environments followed the standard agronomic practices as described for Trial 1 under optimal environments.

### 2.3 Carotenoid analyses

Seed samples used for the carotenoid analyses were produced, as described by Suwarno et al. (2014), by self‐pollinating the first and last two plants per plot in the 196‐hybrid trial involving the 190 PVA and six normal endosperm extra‐early yellow hybrid checks, as well as 20 selected S_8_ plants of PVA inbred lines evaluated under optimal growing conditions at Ikenne and Mokwa in 2016 and 2017. The self‐pollinated ears of the inbred lines and hybrids in each year were harvested per plot, dried under ambient temperature, and shelled (Azmach, Gedil, Menkir, & Spillane, 2013). The seed samples were stored in the long‐term storage facility of IITA at 4°C. Seed samples of the 20 inbred lines used for the diallel cross, along with top yielding 13 PVA hybrids and two checks obtained from composite grains harvested separately from the inbred lines and hybrid trials of each year, were drawn from the long‐term storage. The carotenoids were extracted and quantified at the Food and Nutritional Laboratory of IITA, Ibadan, Nigeria. The High‐Performance Liquid Chromatography (HPLC) method, based on the extraction protocol described by Howe and Tanumihardjo (2006), was employed for the carotenoid analysis. The five carotenoids, β‐carotene (*cis* and trans isomers), α‐carotene, β‐cryptoxanthin, zeaxanthin, and lutein were determined based on calibrations using external standards. Total carotenoids were computed as the sum of concentrations of α‐carotene, β‐carotene, lutein, zeaxanthin, and β‐cryptoxanthin. PVA was computed as the sum of β‐carotene, and half of each of β‐cryptoxanthin and α‐carotene contents, because β‐cryptoxanthin and α‐carotene contribute about 50% of the β‐carotene as PVA according to the US Institute of Medicine (2001). Two independent measurements were taken to represent each sample. In addition to the PVA levels of the hybrids (HPVA) determined by chemical analysis, those of the mid‐parent PVA (MP) were estimated as the average of the sum of PVA levels of parental inbred lines of a specific hybrid.

### 2.4 Field data collection

Data were recorded on DA, DS, ASI, PHT, ear height (EHT), PASP, EASP, root lodging (RL), stalk lodging (SL), husk cover (HUSK), EPP, ear rot (EROT), and grain yield of hybrids evaluated in induced drought and optimal environments in Trial 1 and in low‐N, high‐N, *Striga*‐free, and optimal environments in Trial 2. The STGR was measured at 70 DAP in Trial 1 under drought and in Trial 2 under low‐N stress. Traits assayed for Trials 1 and 2 under artificial *Striga* infestation included DA, DS, ASI, EHT, HUSK, SL, RL, EPP, EASP, EROT, *Striga* damage syndrome ratings at 8 and 10 WAP (SDR1 and SDR2), emerged *Striga* plants at 8 and 10 WAP (ESP1 and ESP2), and grain yield. For details on the observations made on traits and the appropriate scoring scales used in this research, refer to Badu‐Apraku et al. (2015b).

### 2.5 Analysis of data

Combined analysis of variance (ANOVA) was performed for agronomic traits of Trial 1 (genetic study) across year‐location combinations in *Striga*‐infested, drought, and optimal growing environments using the SAS codes for GLM and the RANDOM statement with the TEST option (SAS Institute, 2011). Genotypes were considered fixed effects, whereas test environments, replications, genotype × environment interaction, and all other sources of variation were treated as random effects.

For Trial 2 (RMHT), ANOVA was done separately across stress (*Striga*‐infested and low‐N) and nonstress (*Striga*‐free, high‐N, and optimal) growing environments. The statistical model employed for the combined analysis in the present study has been previously described by Badu‐Apraku et al. (2015b). Broad‐sense heritability (H^2^) of the traits was estimated as the proportion of the phenotypic variance contributed by the genetic variance based on the hybrid means, following the method of Hallauer, Carena, and Filho (2010). Repeatability (*R*) of grain yield and other measured characters was computed for individual environments using the following formula:

$$R=\frac{\sigma_{g}^{2}}{\sigma_{g}^{2}+\left(\sigma_{e}^{2}/r\right)}$$

The standard errors for heritability and repeatability estimates (Hallauer et al., 2010) were computed and used for a pairwise comparison of calculated estimates of the two parameters. Excluding the checks from the analysis of Trial 1, the GCA effects of the PVA parental lines and the SCA effects of F_1_ hybrids, as well as their mean squares under each and across research conditions, were estimated according to Griffing's Method 4 model 1 (fixed effects; Griffing, 1956), using the DIALLEL‐SAS program (Zhang, Kang, & Lamkey, 2005) in SAS software version 9.3 (SAS Institute, 2011). The significance of the GCA and SCA effects were tested using t‐statistic. The square root of the GCA and SCA variances provided an estimate of the standard errors corresponding to their effects (Griffing, 1956). The relative importance of GCA and SCA was examined following the method proposed by Baker (1978), as modified by Hung and Holland (2012). The importance of the combining ability effects was examined by expressing the GCA effects as the ratio of the total genetic effects (i.e., 2GCA + SCA). The closer the ratio to unity (equivalent to 100%), the greater the predictability of hybrid performance based on GCA effects alone (Baker, 1978).

The PVA lines were assigned to the HGs across test environments using the GCA effects of multiple traits (HGCAMT) grouping method (Badu‐Apraku et al., 2013). This was accomplished by standardizing GCA effects of measured traits that showed significant mean squares for genotypes across test environments, and subjecting the dataset to Ward's minimum variance cluster analysis based on the Euclidean distance obtained from HGCAMT via SAS software 9.3 (SAS Institute, 2011). To qualify as a tester, an inbred must (i) have a high, statistically significant positive GCA effect for grain yield, (ii) belong to a heterotic group, and (iii) possess a high per se grain yield (Pswarayi & Vivek, 2008). Single‐cross testers were also identified according to the criteria established by Pswarayi and Vivek (2008), which included (i) parental inbred lines involved in the development of the hybrids must have positive and significant GCA effects for grain yield, (ii) parental lines of hybrids must belong to the same heterotic group, and (iii) the single‐cross hybrid must have a reasonable grain yield.

The ANOVA was performed on plot means of grain yield across test environments to determine whether the genome (G) × environment (E) interaction was significant. For traits with significant G × E interaction mean squares, the genotype main effect plus G × E interaction (GGE) biplot was used to determine the performance and stability of selected top 15, middle five, and worst five PVA maize hybrids, plus five yellow hybrid checks across test environments. All the hybrids in the RMHT were also subjected to GGE biplot analysis. The GGE biplot is a Windows application software that fully automates biplot analysis (Yan, 2001). Information on the GGE biplot program may be found online (www.ggebiplot.com, accessed 13 February 2019). The relationships among traits were investigated using the stepwise multiple regression analyses (SPSS, 2007) and illustrated with sequential path diagrams (Badu‐Apraku, Akinwale, & Oyekunle, 2014; Mohammadi, Prasanna, & Singh, 2003). In this method, HPVA was considered the primary trait, and was regressed on the MP of grain yield and other traits. Traits with significant contributions to HPVA were identified as first order traits. Subsequently, each first order trait was regressed on traits not in the first order category to identify those with significant contributions to HPVA through the first order traits. These were grouped as second order traits. The procedure was continued until all measured traits had been categorized. The standardized *b*‐values of the regression analysis provided an estimate of the path coefficient which was tested for significance using the *t*‐test at .05 probability level (Badu‐Apraku et al., 2014; Mohammadi et al., 2003).

## 3 RESULTS AND DISCUSSION

### 3.1 PVA levels of inbred lines and hybrids

The PVA levels of inbred lines used in this study were significantly different, ranging from 7.88 μg g^−1^ for TZEEIOR 27 to 23.98 μg g^−1^ for TZEEIOR 202 (Table 1) with an overall mean of 10.91 μg g^−1^ and standard error of the mean of .91. Eight (40%) of the lines had PVA values greater than 10 μg g^−1^ and two of them, TZEEIOR 202 (23.98 μg g^−1^) and TZEEIOR 205 (22.56 μg g^−1^), had values significantly higher than the overall mean. Similarly, the hybrids resulting from the crosses of the 20 inbred lines had significantly different PVA values with two of the hybrids, TZEEIOR 197 × TZEEIOR 205 (20.1 μg g^−1^) and TZEEIOR 202 × TZEEIOR 205 (22.7 μg g^−1^), having values much higher than the target of 15 μg g^−1^ proposed by HarvestPlus (Table 1). Here also, relatively few hybrids had PVA values greater than 10 μg g^−1^.

Theoretically, as well as in practice, most traits of maize inbred lines display hybrid vigor or heterosis in single crosses. Results of the inbred lines and their resulting hybrids seemed to deviate from the expected trend that PVA values of hybrids would be higher than the MP value. For the inbred lines and their hybrids summarized in Table 1, only four hybrids had positive heterosis; that is, TZEEIOR 109 × TZEEIOR 197 (1.4%), TZEEIOR 41 × TZEEIOR 97 (2.6%), TZEEIOR 142 × TZEEIOR 250 (20.5%), and TZEEIOR 197 × TZEEIOR 205 (29.5%). All other crosses in Table 1 had negative MP heterosis. From the viewpoints of breeding for improved PVA values, inbred lines TZEEIOR 202 and TZEEIOR 205, and hybrids TZEEIOR 142 × TZEEIOR 250 and TZEEIOR 197 × TZEEIOR 205 were of interest. The inbred lines in this group, TZEEIOR 205 in particular, would likely serve as important sources of favorable alleles for PVA improvement of maize breeding populations. TZEEIOR 205 was one of the parents of the two hybrids with the highest PVA values, and their PVA contents nearly doubled that of the commercial PVA check, TZEEI 58 × TZEE‐Y Pop STR C5 (11.4 μg g^−1^; Table 1). The beneficial alleles in the high PVA parental lines must have been transmitted to the hybrids TZEEIOR 197 × TZEEIOR 205 and TZEEIOR 202 × TZEEIOR 205 which, in turn, displayed high levels of PVA in this study. If further studies, particularly on‐farm trials, confirm the consistency of the performance of the hybrids in contrasting environments, the hybrids would be invaluable in the struggle to overcome hunger and malnutrition in SSA. Although this study did not investigate the stability of PVA content in genotypes from one environment to another, some earlier studies reported that the PVA content of genotypes are not influenced by the environment. For example, Menkir, Liu, White, Maziya‐Dixon, and Rocheford (2008) examined tropical yellow maize inbred lines sampled from four trials in one location and a fifth trial conducted in two locations, and found that carotenoid concentrations of lutein, zeaxanthin, β‐carotene, β‐cryptoxanthin, α‐carotene, and total PVA contents were not strongly affected by the differences in replications or locations or G × E interaction (GEI). In another study conducted by Menkir and Maziya‐Dixon (2004), no significant GEI was obtained for β‐carotene of 17 maize genotypes evaluated in three locations for 2 yr.

### 3.2 ANOVA for agronomic traits of PVA hybrids evaluated under contrasting environments

Environments, genotypes, and GEI sources of variation significantly affected grain yield and most other traits of the PVA hybrids in the *Striga*‐infested, drought, and optimal environments (Table 2). Some traits, such as DA, PHT, EHT, RL, and EROT, were consistently not affected by one or more of the three sources of variation in the three environmental conditions of the study (Table 2). The proportion of total variation due to the environment varied among the field trial conditions. Grain yield, for example, had much larger proportion due to the environment for optimal (45%) than *Striga* (28%) and drought (6%) conditions. Similar values for G were 15, 25, and 37%; and for GEI were 12, 14, and 25% for the three field trial conditions, respectively. The stress environments, *Striga*‐infested in particular, had more traits with nonsignificant G and GEI mean squares than the optimal environmental conditions. However, significant differences occurred among the hybrids for grain yield and some other traits, an indication that real variability existed among the hybrids which could be exploited during selection for these traits under the stress factors (Badu‐Apraku et al. 2015a, 2015b, 2015c). Significant GEI effect detected for grain yield and some other measured traits is also desirable; an implication that PVA hybrids adapted to specific stress and nonstress environments are potentially available in the extra‐early PVA maize germplasm at IITA (Badu‐Apraku, Fakorede, & Lum, 2008). The consistent expression of ASI, PHT, EHT, RL, SL, HUSK, and EROT irrespective of the environments in which the hybrids were tested, if confirmed in further studies, could be an advantage to the breeder to minimize evaluation costs by reducing the number of environments in which data are obtained on these traits for PVA hybrids.

**Table 2:** Mean squares for grain yield and other traits of 190 extra‐early maturing provitamin A (PVA) hybrids evaluated under *Striga*, drought, and optimal conditions in Nigeria during 2015 and 2017 growing seasons

Source	DF a	Yield	DA	DS	ASI	PHT	EHT	RL	SL	HUSK	EASP	EROT	EPP	SDR1	SDR2	ESP1	ESP2
		kg ha^−1^				cm		%									
Striga																	
Block (Rep × E)	52	3692594**	16.5**	9.7**	4.8**	1108.6**	512.5**	50.1**	200.1**	2.4**	2.5**	4.5**	0.09**	2.4**	2.5**	60.0**	98.5*
Rep (E)	2	31147090**	0.9 ns	3.6 ns	2.8 ns	1576.6**	19225.1**	107.0**	696.8**	2.1*	5.1**	69.9**	0.25**	0.3 ns	1.4 ns	217.2**	1695.3**
Entry	195	1726826**	9.5**	6.4**	2.9**	307.2 ns	126.1 ns	26.8 ns	133.7*	0.9**	2.1**	1.9 ns	0.07**	0.9**	1.0**	39.9*	90.9**
E	1	375098444**	9.7 ns	90.3**	7.6 ns	4323.1**	2.0 ns	1754.8**	84.9 ns	133.9**	119.4**	832.7**	20.55**	395.7**	133.9**	42.7 ns	602.0**
Entry × E	195	924529**	6.7**	4.3**	2.2 ns	226.2 ns	115.5 ns	25.1 ns	110.2 ns	0.7 ns	1.0**	1.8 ns	0.05**	0.7*	0.7**	41.2*	83.6*
GCA	19	6558666**	23.1**	12.7**	4.7**	701.8**	390.9**	29.2 ns	265.4**	2.4**	7.3**	2.7 ns	0.21**	1.8**	2.5**	103.6**	326.7**
SCA	170	2000183 ns	11.4 **	7.0**	3.4**	437.7*	173.9 ns	29.1 ns	129.0 ns	1.3**	2.2**	2.0 ns	0.08 ns	1.2**	1.2 ns	40.2 ns	78.0 ns
GCA × E	19	599528 ns	5.8 ns	4.5 ns	1.6 ns	109.1 ns	90.2 ns	15.1 ns	−4.2 ns	0.1 ns	0.6 ns	2.5 ns	0.06 ns	0.2 ns	0.4 ns	35.9 ns	31.1 ns
SCA × E	170	1266920*	8.6 **	6.0**	2.4 ns	279.2 ns	133.0 ns	29.0 ns	127.4 ns	0.8 ns	1.2**	2.1 ns	0.05**	0.8 ns	0.8 ns	43.9 ns	83.1*
ERROR	338	540162	3.88	2.61	1.96	252.69	103.69	26.03	102.63	0.61	0.67	1.69	0.03	0.51	0.52	32.47	62.50
CV, %		38.3	3.4	2.9	71.6	10.1	16.5	58.9	53.5	16.3	15.9	93.3	27.3	16.2	14.9	42.1	60.8
Repeatability		0.48	0.31	0.30	0.27	0.22	0.11	0.03	0.16	0.29	0.53	0.06	0.31	0.27	0.27	0.00	0.09

Drought	DF	Yield	DA	DS	ASI	PHT	EHT	RL	SL	HUSK	PASP	EASP	Ear rot	EPP	STGR		
		kg ha^−1^				cm		%									
Block (Rep × E)	78	1063998**	12.1**	20.0**	6.2**	852.7**	351.8**	100.9**	144.9**	1.3**	1.3**	1.3**	3.6**	0.07**	1.6**	–	–
Rep (E)	3	2096112**	26.5**	27.0**	0.3 ns	5135.4**	2231.4**	48.3 ns	775.5**	11.2**	3.1**	1.1 ns	12.8**	0.06 ns	2.6 ns	–	–
Entry	195	1399500**	17.1**	24.0**	7.0**	366.3**	152.3**	46.2**	145.9**	1.4**	2.1**	2.5**	0.9**	0.14**	1.9**	–	–
E	2	22121161**	2140.0**	152.5**	1610.2**	6169.7**	277.0 ns	2116.5**	17560.8**	355.7**	73.3**	8.1**	1025.4**	0.65**	363.1**	–	–
Entry × E	390	464202**	7.4**	11.7*	5.5**	285.0*	105.5 ns	52.5**	72.0 ns	0.9**	0.6**	0.7**	2.1**	0.04**	0.9 ns	–	–
GCA	19	5971057**	47.0**	70.7**	20.7**	360.9 ns	316.6**	95.5**	463.4**	3.9**	6.6**	8.2**	9.9**	0.44**	7.5**	–	–
SCA	170	1059737**	14.5**	20.1**	6.0 ns	495.0**	177.9**	60.1**	113.2**	1.3 ns	1.8**	2.1**	2.5 ns	0.12**	1.7 ns	–	–
GCA × E	38	556197*	6.2 ns	13.4**	6.6*	347.0 ns	72.1 ns	107.1**	89.5 ns	1.4**	0.8 ns	0.5 ns	2.6*	0.04 ns	1.5*	–	–
SCA × E	340	489170**	8.4**	12.7**	5.8**	367.7 ns	120.6 ns	69.7**	69.4 ns	0.9**	0.7*	0.8**	2.5**	0.05**	1.0 ns	–	–
ERROR	507	281443.40	3.30	6.30	3.80	232.30	101.30	32.40	66.30	0.40	0.50	0.60	1.40	0.03	0.80	–	–
CV, %		42.1	3.5	4.5	49.2	11.2	15.6	63.4	47.1	11.3	12.8	13.8	69.5	25.5	20.0		
Repeatability		0.68	0.58	0.53	0.21	0.25	0.34	0.00	0.52	0.38	0.70	0.73	0.28	0.70	0.54		

Optimal	DF	Yield	DA	DS	ASI	PHT	EHT	RL	SL	HC	PASP	EASP	Ear rot	EPP			
		kg ha^−1^				cm		%									
Block (Rep × E)	104	1951319**	3.8**	5.1**	1.0 ns	654.7**	353.5**	278.7**	50.5**	1.4**	0.8**	5.2 ns	3.6**	0.04**	–	–	–
Rep (E)	4	227382227**	6.7*	31.7**	9.9**	4730.4**	3847.2**	11370.9**	529.8**	6.1**	1.1*	85.7**	2.8 ns	0.11**	–	–	–
Entry	195	4481319**	11.9**	19.9**	2.9**	718.8**	402.9**	185.2**	52.4**	1.4**	1.9**	8.2**	3.0**	0.11**	–	–	–
E	3	875532996**	881.8**	321.0**	383.9**	318584.9**	120777.6**	19418.9**	3050.2**	1493.3**	163.2**	506.9**	520.7**	9.84**	–	–	–
Entry × E	585	1186080**	3.7**	5.4**	1.5**	346.4**	207.5**	142.4**	55.9**	0.8 ns	0.6**	4.8 ns	1.7**	0.04**	–	–	–
GCA	19	20534672**	34.6**	57.4**	6.5**	2580.8**	2319.3**	718.6**	94.1**	4.2 ns	7.3**	19.3**	11.3**	0.40**	–	–	–
SCA	170	3191690 ns	9.9**	15.8**	2.5 ns	643.4 ns	276.6 ns	162.2 ns	53.7**	1.3 ns	1.3**	7.3*	2.5 ns	0.09**	–	–	–
GCA × E	19	6012006**	17.8**	28.4**	6.4 ns	1262.2**	657.2**	1722.8**	237.7**	3.9**	2.8**	19.0**	15.5**	0.22**	–	–	–
SCA × E	170	3699683**	12.1**	17.5**	4.7 ns	1121.3**	646.1**	359.8**	161.4**	2.9**	1.9**	14.7**	5.7**	0.12**	–	–	–
ERROR	676	722235	2.20	2.69	1.05	266.10	148.88	118.87	34.84	0.79	0.41	4.34	1.24	0.02	–	–	–
CV, %		30.3	2.8	3.1	73.8	9.6	15.8	64.7	58.3	19.5	12.4	42.0	96.7	20.7			
Repeatability		0.74	0.70	0.73	0.46	0.53	0.50	0.24	0.00	0.42	0.70	0.41	0.31	0.65			

^a^DF, degrees of freedom; yield, grain yield; DA, days to anthesis; DS, days to silking; ASI, anthesis‐silking interval; PHT, plant height; EHT, ear height; RL, root lodging; SL, stalk lodging; HUSK, husk cover; EASP, ear aspect; EROT, ear rot; EPP, ears per plant; SDR1 and SDR2, *Stringa* damage syndrome ratings at 8 and 10 wk after planting, respectively; ESP1 and ESP2, emerged *Stringa* plants at 8 and 10 wk after planting, respectively; E, environment; GCA, general combining ability; SCA, specific combining ability; CV, coefficient of variance; STGR, stay green characteristic.

*Significant at the .05 probability level.

**Significant at the .01 probability level.

Under optimal conditions, the much larger proportion of total variation caused by the environment (45%) relative to proportions due to G (15%) and GEI (12%) calls for attention. First, the results show that the recommendation that hybrids be tested over multiple environments for several years prior to promotion for release and commercialization (Badu‐Apraku, Fakorede, & Lum, 2007; Ifie, Badu‐Apraku, Gracen, & Danquah, 2015) is also applicable to PVA hybrids. Second, although significant differences observed among the hybrids for all measured traits in this study would facilitate the identification of hybrids with desired attributes (Bhatnagar, Betran, & Rooney, 2004), it seems the environment would greatly regulate the response to selection, an observation similar to that made for some modified‐endosperm opaque‐2 tropical maize inbred lines (Pixley & Bjarnason, 1993). Third, coefficients of variation and repeatability, which are some of the parameters used as indicators of reliability of production estimates (Badu‐Apraku, Fakorede, Menkir, & Sanogo, 2012), varied widely among traits and evaluation conditions (Table 2). Although the grain yield coefficient of variance (CV) was lower and repeatability estimate was higher for optimal, relative to *Striga* and drought environments, the values call for more stringent management conditions to optimize production of PVA hybrids, an indication that the genotype × environment × management interaction is operating in PVA hybrid maize production. However, heritability estimates for grain yield ranged from 30–69% under contrasting environments used in the present study (Supplementary Table 1). This observation, along with CV ≤ 30 for most traits under all environments in this study, indicated that the dataset from the test locations were reliable with minimal or no systematic error. Although the test environments used in the present study were consistent in discriminating among the agronomic traits of the hybrids, the results suggest that the type of environmental condition used by scientists would depend on the breeding strategies and product target.

### 3.3 Combining ability effects

**Figure 1:**
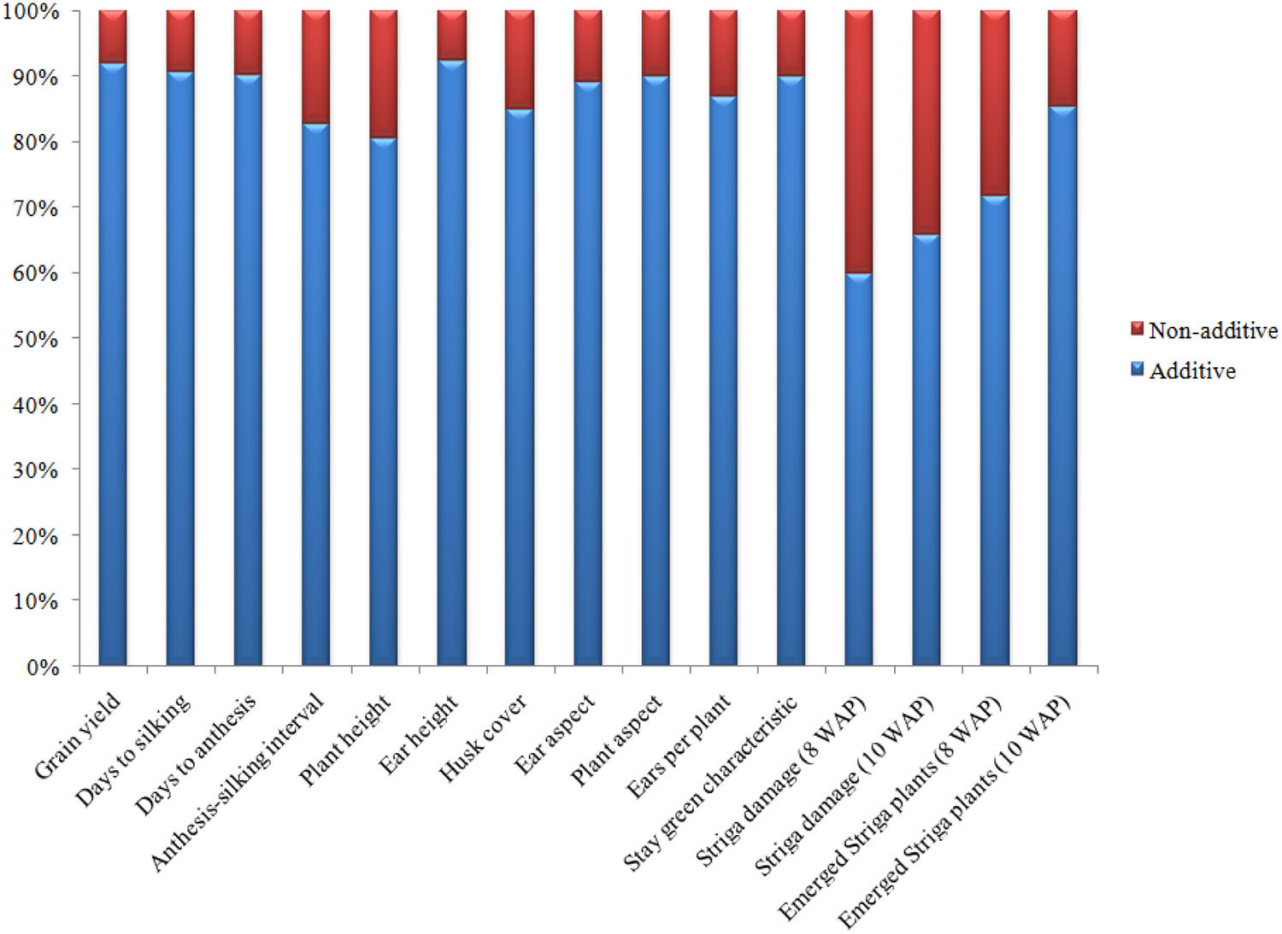
Proportion of additive (lower bar) and non‐additive (upper bar) genetic variances for grain yield and other agronomic traits of 20 extra‐early provitamin A (PVA) inbred lines involved in diallel crosses evaluated across drought, *Striga*‐infested, and rainfed environments in Nigeria, 2015–2017. WAP, weeks after planting

As indicated by significant GCA and SCA mean squares under each evaluation condition, both additive and nonadditive gene actions were involved in the inheritance of most measured traits of the genetic materials evaluated in this study (Table 2). Across all test environments, > 60% of the total genetic effect was attributable to GCA for grain yield and other traits (Figure 1). These results support the general evidence in the literature; that is, additive gene action controls inheritance of most traits of maize, although nonadditive gene action along with environmental effects could also be important, but to a lesser extent. In other words, PVA inbred lines are not different from other inbred lines in terms of quantitative inheritance. Under optimum conditions, GCA × E and SCA × E were also statistically significant for all traits except ASI. This was not the case under the stress environments where relatively few GCA × E and SCA × E interactions were significant. In fact, under *Striga* infestation, GCA × E interaction was not significant for any trait, including grain yield. These results suggested that the inheritance of the PVA maize traits controlled by both additive and nonadditive gene action was not dependent on the environment within each evaluation condition to much appreciable extent (Wegary, Vivek, & Labuschagne, 2013). This is desirable because it indicates that the performance of the hybrids produced from the inbred lines in this study could be reliably predicted based, to a large extent, on the GCA effects alone (Baker, 1978). Contrarily, significant GCA × E and SCA × E interaction effects for most traits under optimal conditions, and some of the traits under the stress conditions indicated that mode of inheritance of the traits could vary under different environmental conditions, as observed in earlier studies (Badu‐Apraku, Lum, Akinwale, & Oyekunle, 2011b).

### 3.4 GCA effects of inbred lines

Among the lines evaluated in the study, eight (TZEEIOR 97, TZEEIOR 142, TZEEIOR 197, TZEEIOR 202, TZEEIOR 205, TZEEIOR 209, TZEEIOR 250, and TZEEIOR 251) had significant positive GCA effects for grain yield across test environments and mostly under each environmental condition (Table 3). These inbred lines are likely to produce high grain yield in hybrid combinations (Badu‐Apraku et al., 2015a). However, none of the inbred lines consistently had significant GCA effects for all the other traits assayed in the study. The effects were significant for some traits, ranging from two for TZEEIOR 209 to nine for TZEEIOR 197 and TZEEIOR 250 (Table 3). Extra‐early maturing PVA inbred lines tolerant to drought, and those resistant to *Striga* infestation were also identified for the first time in this study. Inbreds TZEEIOR 97, TZEEIOR 197, TZEEIOR 250, and TZEEIOR 251 displayed significant negative effects for STGR across drought environments. Hybrids produced in crosses involving lines TZEEIOR 97, TZEEIOR 197, TZEEIOR 250, and TZEEIOR 251 that had significant negative GCA effects for STGR under drought environments would be characterized by delayed leaf senescence, as noted by Badu‐Apraku et al. (2015b). Similarly, hybrids involving lines TZEEIOR 251 and TZEEIOR 197 which had significant negative GCA effects would have low *Striga* damage at 8 and/or 10 WAP under *Striga*‐infested environments. For number of emerged *Striga* plants at 8 and/or 10 WAP in *Striga*‐infested environments, inbred lines TZEEIOR 197, TZEEIOR 30, TZEEIOR 140, and TZEEIOR 142 had negative GCA effects, an indication that they possessed beneficial alleles for *Striga* resistance which could be passed on to the progenies (Badu‐Apraku et al., 2015a, 2015b). It is noteworthy that the inbreds TZEEIOR 97 and TZEEIOR 251 had significant positive GCA effects for grain yield across environments as well as significant negative GCA effects for STGR under drought. The inbred TZEEIOR 251 had significant and positive effects of GCA for grain yield across test environments and significant negative GCA effects for STGR in drought as well as in *Striga*‐infested environments. The performance of inbred line TZEEIOR 197 was particularly striking. Across all environments, this line showed significant positive GCA effects for grain yield, but significant negative effects for STGR under drought as well as for SDR2 and ESP1 under *Striga*‐infested environments. This suggested that TZEEIOR 197 could serve as a potential source of beneficial alleles for improved grain yield, drought tolerance, and *Striga* resistance and/or tolerance in PVA hybrids. The line could also be introgressed into consumer‐acceptable tropical PVA germplasm that are otherwise susceptible to drought and *Striga* infestation. In addition, inbred line TZEEIOR 197 may be a potentially good tester for PVA single‐cross hybrid production.

**Table 3:** General combining ability (GCA) effects of grain yield and other agronomic traits of 20 extra‐early maturing provitamin A (PVA) maize inbreds evaluated across three drought, two *Striga*‐infested and four optimal environments in Nigeria, 2015–2017

	Grain yield															
Inbred	Drought	*Striga*‐infested	Optimal	Across environments	DA^a^	DS	ASI	HUSK	PASP	EASP	EPP	STGR	SDR1	SDR2	ESP1	ESP2
	kg ha^−1^															
TZEEIOR 22	−226**	−543**	−362*	−383**	0.81**	0.89**	0.13 ns	0.22*	0.28**	0.53**	−0.077**	0.31 ns	0.20 ns	0.28*	−0.08 ns	−1.01 ns
TZEEIOR 24	−351**	−184	−404**	−346**	0.57**	0.73**	0.17 ns	0.15 ns	0.26*8	0.24*	−0.050**	0.36*	0.06 ns	0.03 ns	−0.36 ns	0.35 ns
TZEEIOR 26	−114	−301*	−161	−219*	−0.05 ns	0.09 ns	0.20 ns	0.17 ns	0.26**	0.15 ns	−0.055**	0.56**	0.14 ns	0.14 ns	1.27 ns	2.58*
TZEEIOR 27	−267**	−292*	−307*	−299**	0.15 ns	0.26 ns	0.15 ns	0.08 ns	0.28**	0.27*	−0.051**	0.20 ns	0.13 ns	0.03 ns	−0.09 ns	−2.01 ns
TZEEIOR 28	−283**	−313*	−369*	−333**	0.05 ns	0.21 ns	0.16 ns	0.07 ns	0.15*	0.36**	−0.066**	−0.03 ns	0.18 ns	0.31**	0.20 ns	−0.13 ns
TZEEIOR 30	106	−98	−529**	−251**	0.62**	0.63**	0.02 ns	−0.01 ns	0.20**	0.17 ns	−0.009 ns	−0.28 ns	0.10 ns	−0.04 ns	−1.30 ns	−2.92**
TZEEIOR 41	−296**	−36	−405**	−281**	−0.06 ns	0.25 ns	0.33**	0.07 ns	0.17**	0.24*	−0.066**	−0.01 ns	−0.15 ns	−0.02 ns	−0.62 ns	−0.38 ns
TZEEIOR 45	−223**	−319*	−628**	−440**	0.51**	0.63**	0.11 ns	0.17 ns	0.31**	0.33**	−0.049**	0.21 ns	0.20 ns	0.17 ns	−0.55 ns	−1.53 ns
TZEEIOR 97	180**	85	345*	240*	0.48**	0.34*	−0.13 ns	−0.02 ns	−0.17*	−0.02 ns	0.013 ns	−0.35*	0.03 ns	−0.04 ns	0.57 ns	1.94 ns
TZEEIOR 109	463**	256	21	189 ns	−0.66**	−0.91**	−0.29*	−0.13 ns	−0.24**	−0.26*	0.015 ns	−0.10 ns	−0.01 ns	−0.08 ns	0.56 ns	0.20 ns
TZEEIOR 140	−39	185	323*	176 ns	0.24 ns	0.37*	0.19 ns	−0.07 ns	−0.09 ns	−0.18 ns	0.047**	0.21 ns	−0.26 ns	−0.19 ns	−1.13 ns	−2.66*
TZEEIOR 142	91	401**	493**	360**	0.51**	0.39*	−0.13 ns	−0.18 ns	−0.24**	−0.38**	0.036*	0.06 ns	−0.18 ns	−0.08 ns	−1.37 ns	−2.55*
TZEEIOR 197	343**	655**	270	401**	−0.50**	−0.37*	0.14 ns	−0.39 **	−0.17*	−0.50**	0.072**	−0.37*	−0.18 ns	−0.44**	−2.08*	−1.20 ns
TZEEIOR 202	34	−172	433**	199*	0.09 ns	0.17 ns	0.03 ns	−0.22 *	−0.35**	−0.33**	0.063**	−0.12 ns	0.09 ns	−0.02 ns	−0.51 ns	0.38 ns
TZEEIOR 205	259**	16	646**	405**	−0.27 ns	−0.43*	−0.19 ns	−0.06 ns	−0.25**	−0.36**	0.066**	−0.08 ns	0.09 ns	0.17 ns	1.81*	4.30*
TZEEIOR 209	−83	216	305*	195*	−0.54**	−0.70**	−0.23 ns	−0.06 ns	−0.09 ns	−0.13 ns	0.024 ns	0.03 ns	−0.15 ns	−0.15 ns	1.14 ns	0.26 ns
TZEEIOR 233	80	7	−68	−14 ns	−0.43*	−0.69**	−0.28*	0.20*	−0.03 ns	0.03 ns	0.010 ns	−0.12 ns	−0.03 ns	0.19 ns	2.77**	4.67**
TZEEIOR 234	−112	−162	5	−61 ns	−0.53**	−0.45**	0.02 ns	0.09 ns	−0.01 ns	0.30**	−0.011 ns	0.25 ns	0.10 ns	0.12 ns	−1.32 ns	−1.65 ns
TZEEIOR 250	221**	432**	151	241*	−0.44**	−0.74**	−0.28*	−0.08 ns	−0.13*	−0.23*	0.042**	−0.37*	−0.34*	−0.29*	0.23 ns	1.10 ns
TZEEIOR 251	216**	167	239	221*	−0.55**	−0.68**	−0.09 ns	0.00 ns	−0.15*	−0.23*	0.044**	−0.35*	−0.01 ns	−0.12 ns	0.87 ns	0.26 ns

*Significant at the .05 probability level.

**Significant at the .01 probability level.

a DA, days to anthesis; DS, days to silking; ASI, anthesis‐silking interval; HUSK, husk cover; PASP, plant aspect; EASP, ear aspect; EPP, ears per plant; STGR, stay green characteristic; SDR1 and SDR2, *Stringa* damage syndrome ratings at 8 and 10 wk after planting, respectively; ESP1 and ESP2, emerged *Stringa* plants at 8 and 10 wk after planting, respectively; ns, nonsignificant.

### 3.5 Heterotic groups and identification of testers

The HGCAMT method (Badu‐Apraku et al., 2013) assigned the inbreds into two HGs across environments at 40% level of dissimilarity (i.e., R‐squared value of 40%), with 11 and nine of the lines in HG 1 and HG 2, respectively (Table 4; Supplementary Figure 1). The placement of the inbred lines into two heterotic groups increased the chances of developing high‐yielding hybrids through inter‐mating of inbred lines belonging to opposing HGs. The inbred TZEEIOR 97 was identified as tester for HG 1 and TZEEIOR 197 for HG 2, while TZEEIOR 197 × TZEEIOR 205 was identified as single‐cross tester for HG 2. The identification of inbred testers TZEEIOR 97 and TZEEIOR 197 for HGs 1 and 2 would not only fast‐track the development of outstanding hybrids but also support a conservative approach to hybrid development, as testers identified for each HG could be crossed to lines of opposing HGs. Of interest was the inbred TZEEIOR 197 which was identified as possessing genes for high grain yield, drought tolerance, and *Striga* resistance and/or tolerance, and as one of the new inbred testers. This inbred would definitely be invaluable in the development of high yielding, multiple‐stress tolerant PVA hybrids for commercialization in SSA.

**Table 4:** Heterotic groups of 20 extra‐early maturing provitamin A (PVA) maize inbred lines classified with the HGCAMT methods across eight environments in Nigeria, 2015–2017

Group 1	Group 2
TZEEIOR 22, TZEEIOR 24, TZEEIOR 26, TZEEIOR 27, TZEEIOR 28, TZEEIOR 41, TZEEIOR 45, TZEEIOR 30, TZEEIOR 97, TZEEIOR 233 and TZEEIOR 234	TZEEIOR 109, TZEEIOR 197, TZEEIOR 209, TZEEIOR 250, TZEEIOR 251, TZEEIOR 140, TZEEIOR 142, TZEEIOR 202 and TZEEIOR 205

### 3.6 Performance and stability of provitamin a hybrids across multiple environments

**Figure 2:**
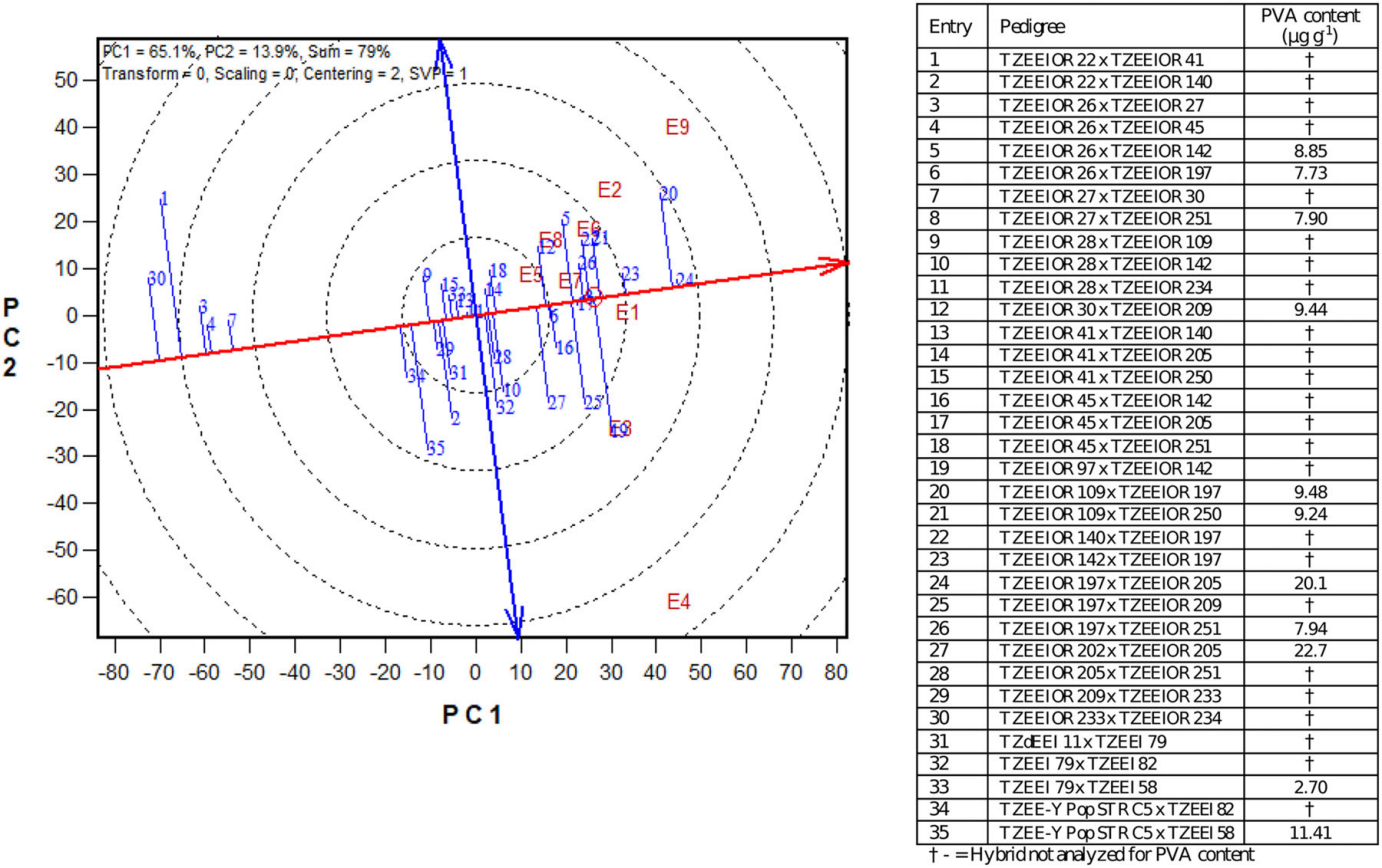
The “mean vs. stability” view of the genotype main effect plus genotype × environment interaction biplot, based on a genotype × environment yield data of selected top 25, worst five extra‐early provitamin A (PVA) hybrids, plus five checks from Trial 1 across nine environments in Nigeria, 2015–2017. PC1 and PC2, principle component 1 and 2, respectively; E1, Ikenne well‐watered, 2016; E2, Mokwa optimal, 2016; E3, Ikenne well‐watered, 2017; E4, Bagauda optimal, 2017; E5, Ikenne drought, 2015; E6, Ikenne drought, 2016; E7, Ikenne drought, 2017; E8, Mokwa *Striga*‐infested, 2016; E9, Mokwa *Striga*‐infested, 2017

**Figure 3:**
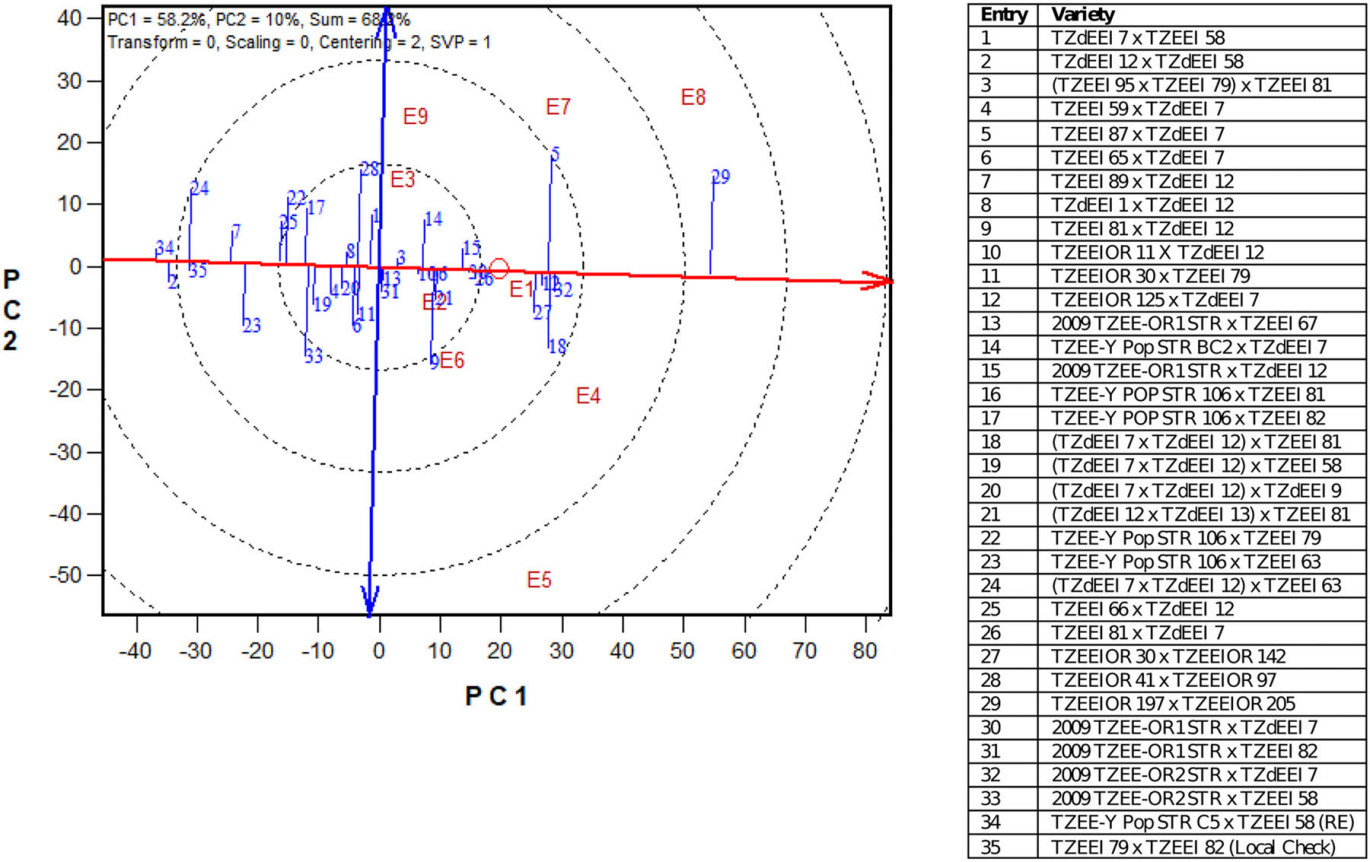
The “mean vs. stability” view of the genotype main effect plus genotype × environment interaction biplot based on a genotype × environment yield data of 33 yellow and provitamin A (PVA) hybrids plus two yellow hybrid checks in the regional trial (Trial 2), across nine environments in Nigeria in 2018. E1, Ife low‐N; E2, Mokwa low‐N; E3, Mokwa *Striga*‐infested; E4, Ikenne Optimal; E5, Ife High‐N; E6, Mokwa High N; E7, Bagauda Optimal; E8, Zaria Optimal; E9, Mokwa *Striga* free

In the GGE biplot view, the thick single‐arrow red line passing through the biplot origin and the average tester is referred to as the average‐tester coordinate axis (ATC). The double‐arrow line (ATC ordinate) separates hybrids with yield less than the average (to the left side of the line) from those with yield greater than the mean (to the right side of the line; Figure 2, 3). The average performance of a hybrid is approximated by the projection of its marker on the ATC. The stability of the hybrids is measured by the projection onto the ATC *y*‐axis single‐arrow line (ATC abscissa). The shorter the absolute length of the projection of the hybrid, the more stable it is (Yan, Kang, Ma, Woods, & Cornelius, 2007). In the GGE biplot, hybrid TZEEIOR 109 × TZEEIOR 197 was promising in terms of grain yield but relatively less stable (Figure 2). The PVA hybrid TZEEIOR 142 × TZEEIOR 197, which ranked third in grain yield, was very stable across test environments. However, hybrid TZEEIOR 197 × TZEEIOR 205 in the genetic study had the highest above mean grain yield as well as the shortest projection onto the average‐tester coordinate *y*‐axis and was, therefore, the highest yielding and most stable hybrid across test environments (Figure 2). Based on the genetic studies, this hybrid was identified as a single‐cross tester for HG 2 and had high PVA content of 20.1 μg g^−1^, which exceeded that of TZEE‐Y Pop STR C5 × TZEEI 58 (commercial check) with 11.5 μg g^−1^, as well as the breeding target of 15 μg g^−1^ set by the HarvestPlus Challenge Program (Badu‐Apraku, Talabi, Gedil, & Garcia‐Oliveira, 2019). The GGE biplot also revealed that TZEEIOR 197 × TZEEIOR 205 was the top‐most yielding extra‐early PVA hybrid in the Regional Trial across environments, but relatively unstable. This hybrid should be tested for agronomic performance on‐farm and promoted for commercialization, since it qualifies as candidate replacement for already existing hybrids in the public domain, to mitigate malnutrition and food insecurity in SSA.

### 3.7 ANOVA and performance of extra‐early yellow and PVA hybrids in RMHT under contrasting environments

Results of ANOVA for extra‐early yellow and PVA hybrids evaluated in RMHT revealed significant differences among the hybrids for all measured agronomic traits across stress and nonstress environments, except for SDR2 under *Striga* infestation (Table 5). The environment effects were significant for all measured traits assayed separately across stress and nonstress environments. In contrast, hybrid × environment interaction effects were significant for grain yield and STGR under stress and for DS, ASI, HUSK, PASP, and EASP across nonstress environments. Furthermore, the results revealed TZEEIOR 197 × TZEEIOR 205 as the highest yielding hybrid across stress (3554 kg ha^−1^) and nonstress (5655 kg ha^−1^) environments, out‐yielding the commercial PVA check, TZEEI 58 × TZEE‐Y STR C5 by 67 and 61%, respectively. In addition, TZEEIOR 197 × TZEEIOR 205 was superior to the commercial hybrid check in terms of HUSK, STGR, and SDR2 under stress, and PASP, EASP, and EPP across stress and nonstress environments.

**Table 5:** Analysis of variance and summary statistics for measured traits of extra‐early yellow and provitamin A (PVA) hybrids evaluated across stress (*Striga*‐infested and low‐N) and nonstress environments in Nigeria, 2018

		Grain yield		DS b		ASI		PHT		HUSK		PASP		EASP		EPP		STGR		
Entry	Variety	STR	NSTR	STR	NSTR	STR	NSTR	STR	NSTR	STR	NSTR	STR	NSTR	STR	NSTR	STR	NSTR	(10 WAP)	SDR2	ESP2
		kg ha^−1^						cm												
29	TZEEIOR 197 × TZEEIOR 205	3554	5655	56	54	3	2	169	188	3	4	4	4	4	4	0.9	0.9	3	5	56
30	2009 TZEE‐OR1 STR × TZdEEI 7	2781	4923	54	52	3	1	161	175	4	4	4	5	5	4	0.8	0.9	3	6	106
10	TZEEIOR 11 × TZdEEI 12	2779	4629	53	52	1	0	151	173	4	4	4	4	4	5	0.8	0.9	4	5	61
13	2009 TZEE‐OR1 STR × TZEEI 67	2723	4467	54	54	1	1	165	180	4	4	4	5	4	4	0.8	0.9	3	5	98
15	2009 TZEE‐OR1 STR × TZdEEI 12	2718	4781	54	52	2	1	158	179	4	4	5	4	5	4	0.9	0.9	3	5	65
12	TZEEIOR 125 × TZdEEI 7	2632	5302	55	53	1	1	142	177	4	4	4	4	5	4	0.8	1.0	3	5	98
9	TZEEI 81 × TZdEEI 12	2627	4848	53	52	2	2	149	173	4	4	5	5	5	4	0.8	0.9	3	5	67
11	TZEEIOR 30 × TZEEI 79	2566	4558	53	52	1	1	163	175	4	4	4	5	4	4	0.9	1.0	3	5	59
3	(TZEEI 95 × TZEEI 79) × TZEEI 81	2530	4771	52	51	2	1	151	172	4	4	5	5	4	4	0.9	0.9	3	5	49
32	2009 TZEE‐OR2 STR × TZdEEI 7	2509	5350	53	53	2	1	148	172	4	4	4	4	5	4	0.8	0.9	3	6	91
6	TZEEI 65 × TZdEEI 7	2506	4459	52	51	2	1	142	174	4	4	4	5	5	4	0.9	0.9	3	6	79
18	(TZdEEI 7 × TZdEEI 12) × TZEEI 81	2484	5158	54	52	3	1	150	178	4	4	4	5	4	4	0.7	0.9	3	5	65
26	TZEEI 81 × TZdEEI 7	2452	4882	53	51	2	1	155	176	4	4	5	5	4	4	0.8	0.9	3	5	74
1	TZdEEI 7 × TZEEI 58	2440	4533	52	51	1	1	149	183	4	4	5	5	5	4	0.7	0.9	3	5	84
		kg ha^−1^						cm												
22	TZEE‐Y Pop STR 106 × TZEEI 79	2413	4124	54	52	2	1	172	187	4	4	5	5	5	4	0.8	0.9	4	5	49
5	TZEEI 87 × TZdEEI 7	2407	5295	53	51	2	1	148	177	3	3	4	4	4	4	0.9	1.0	2	5	74
8	TZdEEI 1 × TZdEEI 12	2406	4135	54	52	2	1	158	181	4	4	4	5	5	5	0.8	1.0	4	5	33
25	TZEEI 66 × TZdEEI 12	2395	4127	54	52	2	1	167	179	4	4	5	5	5	5	0.8	0.9	3	5	51
27	TZEEIOR 30 × TZEEIOR 142	2393	5205	56	55	2	2	161	189	4	4	5	4	5	4	0.9	0.9	3	5	48
14	TZEE‐Y Pop STR BC2 × TZdEEI 7	2380	4659	52	52	1	1	157	172	4	4	5	5	5	4	0.8	0.9	3	5	78
33	2009 TZEE‐OR2 STR × TZEEI 58	2376	4251	53	52	1	1	173	187	4	4	4	4	4	5	0.7	0.8	3	6	57
21	(TZdEEI 12 × TZdEEI 13) × TZEEI 81	2322	4775	54	53	2	1	162	173	4	4	5	5	5	4	0.8	0.9	4	6	83
7	TZEEI 89 × TZdEEI 12	2295	3773	52	50	2	1	150	163	4	4	5	5	5	5	0.9	0.9	3	6	37
23	TZEE‐Y Pop STR 106 × TZEEI 63	2278	3847	53	51	1	1	148	172	4	4	4	5	5	5	0.7	0.9	3	6	76
24	(TZdEEI 7 × TZdEEI 12) × TZEEI 63	2231	3812	52	51	1	1	155	169	4	4	5	5	5	5	0.8	0.9	3	6	53
17	TZEE‐Y POP STR 106 × TZEEI 82	2200	4409	53	50	1	0	167	178	4	4	5	5	5	4	0.8	1.0	3	5	79
4	TZEEI 59 × TZdEEI 7	2157	4200	53	52	2	1	144	164	4	4	5	5	5	5	0.8	0.9	3	6	55
		kg ha^−1^						cm												
34	TZEE‐Y Pop STR C5 × TZEEI 58 (RE)	2122	3522	53	52	2	1	167	183	4	4	5	5	5	5	0.7	0.8	4	6	61
19	(TZdEEI 7 × TZdEEI 12) × TZEEI 58	2119	4299	52	50	2	1	163	180	4	4	4	5	5	5	0.8	0.9	4	5	55
16	TZEE‐Y POP STR 106 × TZEEI 81	2073	4546	55	53	3	1	156	181	4	4	5	5	5	4	0.7	0.9	3	6	42
20	(TZdEEI 7 × TZdEEI 12) × TZdEEI 9	2022	4486	53	52	1	1	145	175	4	4	4	4	5	5	0.8	0.9	3	5	90
35	TZEEI 79 × TZEEI 82 (Local Check)	1996	3723	54	52	2	1	164	179	4	4	5	5	5	5	0.8	0.9	3	6	88
2	TZdEEI 12 × TZdEEI 58	1992	3791	52	50	1	0	143	166	4	4	5	5	5	5	0.9	0.9	4	5	52
28	TZEEIOR 41 × TZEEIOR 97	1866	4371	56	54	3	2	155	184	5	4	6	5	6	4	0.6	0.8	4	5	63
31	2009 TZEE‐OR1 STR × TZEEI 82	1857	4648	53	53	1	1	157	184	4	5	5	5	5	4	0.7	0.9	3	6	93
GRAND MEAN	2389	4523	53	52	2	1	156	177	4	4	5	5	5	4	0.8	1	3	5	68	
LSD, 5%		473	452	1	1	1	1	15	10	0	0	1	0	1	0	0.1	0.1	1	1	32
CV, %		21	15	3	3	72	103	10	9	13	15	12	10	14	16	14	12	16	13	29
		kg ha^−1^						cm												
*P*‐value for G	**	**	**	**	**	**	**	**	**	**	**	**	**	**	**	**	**	ns	*	
*P*‐value for E	**	**	**	**	**	**	**	**	**	**	**	**	**	**	**	**	**			
*P*‐value for G × E	**	**	ns	*	ns	**	ns	ns	ns	**	ns	**	ns	**	ns	ns	**			

^a^DS, days to silking; ASI, anthesis‐silking interval; PHT, plant height; HUSK, husk cover; PASP, plant aspect; EASP, ear aspect; EPP, ears per plant; STGR, stay green characteristic, SDR2, *Stringa* damage syndrome ratings 10 wk after planting; ESP2, emerged *Stringa* plants 10 wk after planting; STR, stressed; NSTR, nonstressed; WAP, wk after planting; LSD, least significant difference; CV, coefficient of variance; G, genome; ns, nonsignificant; E, environment.

^*^Significant at the .05 probability level.

^**^Significant at the .01 probability level.

The GGE biplot revealed TZEEIOR 197 × TZEEIOR 205 in the RMHT as having the most outstanding above mean grain yield performance and was therefore considered the highest yielding hybrid across test environments (Figure 3). However, the hybrid had a long projection onto the average‐tester coordinate *y*‐axis. Other promising hybrids in terms of grain yield stability across test environments included TZEEIOR 125 × TZdEEI 7 and 2009 TZEE‐OR2 STR × TZdEEI 7.

### 3.8 Stepwise multiple regression and path analysis

**Figure 4:**
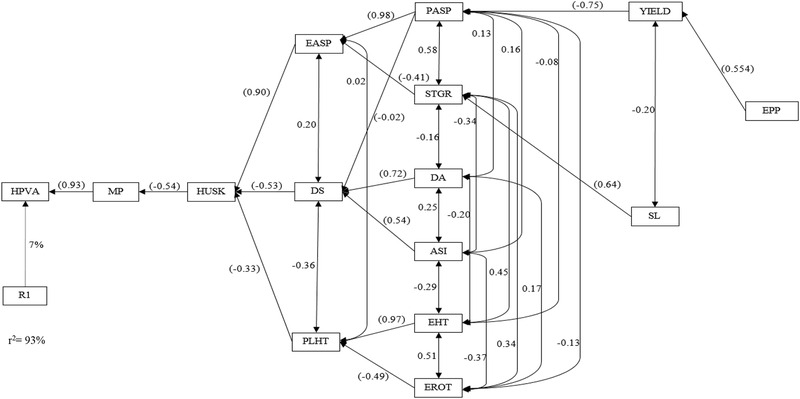
Path analysis model diagram showing causal relationships of hybrid provitamin A (PVA) levels, mid‐parent PVA levels, and other measured traits of PVA diallel crosses evaluated under managed drought stress at Ikenne during the 2015–2016 and 2016–2017 dry seasons. Values in parenthesis are direct path coefficients and other values are correlation coefficients. R1, residual effects; ASI, anthesis–silking interval; DA, days to 50% anthesis; DS, days to 50% silking; EASP, ear aspect; EPP, ears per plant; HPVA, hybrid pro‐vitamin A; HUSK, husk cover; MP, mid‐parent pro‐vitamin A; PASP, plant aspect; PHT, plant height; STGR, stay green characteristic; RL, root lodging; SL, stalk lodging

Information on the interrelationships among traits plays a key role in the choice of secondary traits a breeder would consider for inclusion in the selection index. In the present study, causal relationships among the hybrid PVA levels, MP levels, grain yield, and other measured agronomic traits were illustrated using stepwise regression as well as path analyses under *Striga*‐infested and drought environments (Mohammadi et al., 2003; Talabi et al., 2017). The stepwise multiple regression analysis revealed MP as the sole trait in the first order category accounting for 93% of the observable variation in the PVA levels of hybrids under managed drought environments (Figure 4). This implied that the PVA content of a hybrid is largely dependent on those of the parental lines used to develop it. Husk cover was the only second order trait while EASP, DS, and PHT fell in the third order category of traits contributing to the variation in the hybrid PVA levels. Plant aspect, STGR, DA, ASI, EHT, and EROT were categorized as fourth order traits, while grain yield and SL were classified as fifth order traits. Ears per plant was in the sixth order category of the traits.

**Figure 5:**
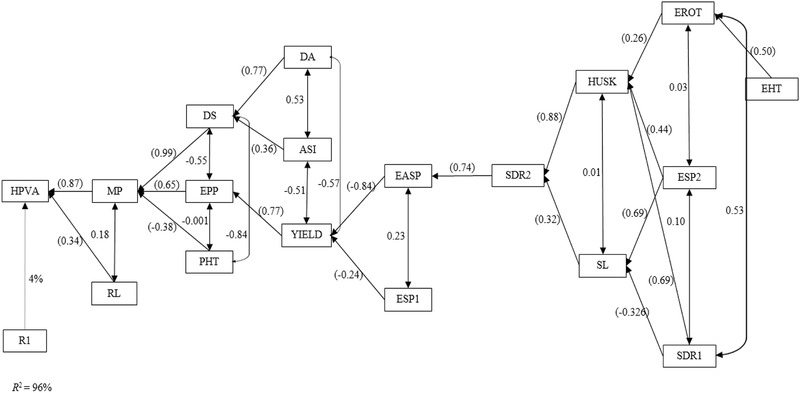
Path analysis model diagram showing causal relationships of hybrid provitamin A (PVA) levels, mid‐parent PVA levels, and other measured traits of PVA diallel crosses evaluated under artificial *Striga* infestation at Mokwa, during the 2016 and 2017 growing seasons. Values in parenthesis are direct path coefficients and other values are correlation coefficients. R1, residual effects; ASI, anthesis–silking interval; DA, days to 50% anthesis; DS, days to 50% silking; EASP, ear aspect; EPP, ears per plant; ESP1 and ESP2, emerged *Striga* plants (8 and 10 WAP, respectively); HUSK, husk cover; PHT, plant height; RL, root lodging; SDR1 and SDR2, *Striga* damage (8 and 10 WAP, respectively)

Across *Striga*‐infested environments, MP and RL were identified as the first order traits explaining about 96% of the observable differences in the hybrid PVA levels (Figure 5). This implied that MP and RL were the primary traits that influenced the HPVA under artificial *Striga*‐infested environments. About 96% of the variation could be attributed to these traits, indicating that the PVA content of a hybrid is a function of the PVA levels of the parental inbred lines as well as the lodging resistance of the PVA hybrids under artificial *Striga*‐infested environments. The traits in the second order category included DS, EPP, and PHT. Days to anthesis, ASI, and grain yield were classified as third order traits, while EASP and ESP1 fell in the fourth order. *Striga* damage syndrome rating at 10 WAP was the only trait in the fifth order, whereas HUSK and SL were among sixth order traits. The seventh order category comprised EROT, ESP2, and SDR1. Ear height was the sole trait in the eighth order under *Striga*‐infested environments. The identification of grain yield as fifth and third order traits under induced‐drought and *Striga*‐infested environments, respectively, implied that the PVA level of hybrids is independent of their yield performance. Thus, simultaneous selection for high grain yield and elevated PVA levels would suffice when the development of hybrids with these characteristics is the goal of a breeding program.

## 4 SUMMARY AND CONCLUSIONS

When the HarvestPlus Challenge Program was initiated, the yellow kernel maize, on average, had 1.5 μg g^−1^ PVA content while the orange color maize had 3–8 μg g^−1^ (HarvestPlus, 2014). Maize breeders then considered maize germplasm with orange kernels as a possible source of PVA and focused attention on the materials for PVA improvement. By implication, PVA genes must have been present in the landraces, although in low frequencies at best. The WECAMAN program subjected the populations developed from the composite of the landraces to genetic enhancement of resistance and/or tolerance to drought, *Striga*, and low‐N, along with improved quality protein and PVA content, both of which are more recent projects. Stress‐tolerant maize varieties that have relatively high nutritious value are now being released to farmers in SSA. The genetic studies and breeding efforts for improved PVA that are presented in this paper were based on the orange kernel materials. Indeed, inbred lines with relatively high PVA have been developed and used as parent materials for hybrids. Among the inbred lines are TZEEIOR 205 and TZEEIOR 202 with high PVA levels of 22.56 and 23.98 μg g^−1^, respectively, which exceeded the target of 15 μg g^−1^ established by the HarvestPlus Challenge Program by 50% and 60%, respectively. These PVA inbred lines could be used as beneficial alleles for improvement of PVA levels of tropical breeding populations or for introgression into other PVA lines through backcrossing. The identification of TZEEIOR 97 and TZEEIOR 197 as PVA inbred testers and TZEEIOR 197 × TZEEIOR 205 as PVA single‐cross tester from this study will fast‐track the development of outstanding PVA single, three‐way, and top‐cross hybrids for commercialization in SSA. Inbred TZEEIOR 197 could serve as an important source of beneficial alleles for improving yield, resilience to drought and *Striga* based on its outstanding combining abilities for grain yield, SDR2, number of ESP2, and STGR under the respective research environments. The PVA hybrid TZEEIOR 197 × TZEEIOR 205 (PVA of 20.1 μg g^−1^), which was identified as the single‐cross tester with outstanding performance both in genetic studies and RMHT, should be extensively evaluated and commercialized to combat food insecurity and malnutrition in SSA. The PVA levels of hybrids were independent of their yield potential suggesting that simultaneous selection for high yield and elevated PVA levels would suffice. Meanwhile, several orange colored open‐pollinated varieties and hybrids with relatively high PVA have been released for commercialization in SSA (HarvestPlus, 2014).

## CONFLICT OF INTEREST

The authors declare that the research was conducted in the absence of any commercial or financial relationships that could be construed as a potential conflict of interest.

## AUTHOR CONTRIBUTIONS

BB developed the genetic materials, conceived, designed and executed the experiment. AT assisted in the execution of the experiment, analyzed the data, and assisted in the drafting of the manuscript. MO contributed to the development of the genetic material and reviewed the manuscript. MA contributed to the development of the genetic material and execution of the experiment. MF, AFL, PR, GA, and JOT reviewed the manuscript. All authors agreed to the final version of the manuscript.

## Supporting information

Supplemental Figure 1. Dendrogram of 20 extra‐early maturing PVA inbred lines constructed from HGCAMT using Ward's minimum variance cluster analysis method across drought, Striga‐infested and optimal environments in Nigeria, 2015‐2017.

Supplemental Table 1. Treatments and heritability estimates of test environments in Nigeria, 2015 to 2017.
