# Supplementary material for: Genetic studies of extra‐early provitamin‐A maize inbred lines and their hybrids in multiple environments
Source: Crop Sci. 2020 May 5;60(3):1325–45. doi: 10.1002/csc2.20071 (PMC7318638; doi:10.1002/csc2.20071)
Supplement: Supplementary file 1 — Supplemental Figure 1. Dendrogram of 20 extra‐early maturing PVA inbred lines constructed from HGCAMT using Ward's minimum variance cluster analysis method across drought, Striga‐infested and optimal environments in Nigeria, 2015‐2017. [file CSC2-60-1325-s001.pdf]

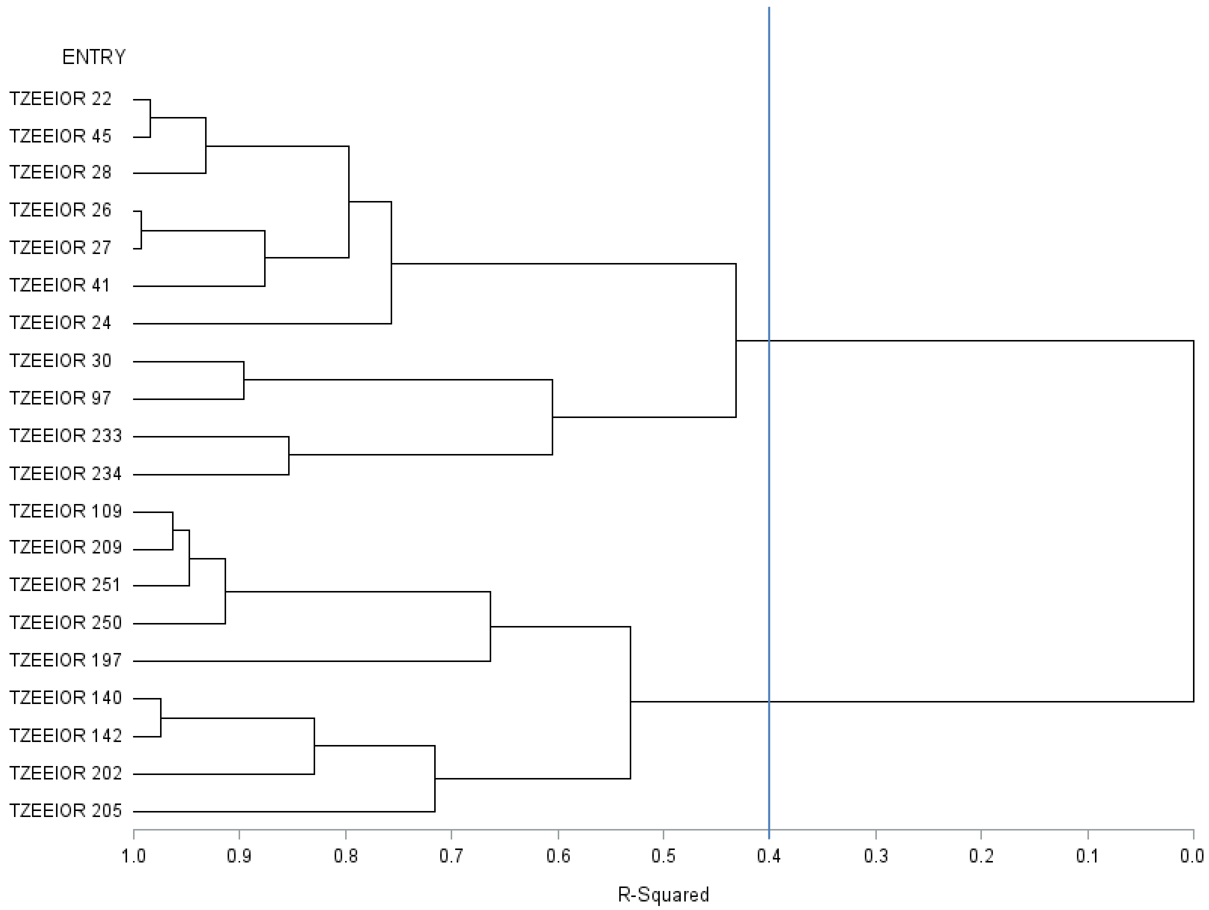

Supplemental Figure 1. Dendrogram of 20 extra-early maturing PVA inbred lines constructed from HGCAMT using Ward's minimum variance cluster analysis method across drought, *Striga*-infested and optimal environments in Nigeria, 2015-2017.
