# Supplementary material for: Genetic studies of extra‐early provitamin‐A maize inbred lines and their hybrids in multiple environments
Source: Crop Sci. 2020 May 5;60(3):1325–45. doi: 10.1002/csc2.20071 (PMC7318638; doi:10.1002/csc2.20071)
Supplement: Supplementary file 2 — Supplemental Table 1. Treatments and heritability estimates of test environments in Nigeria, 2015 to 2017. [file CSC2-60-1325-s002.pdf]

Supplemental Table 1. Treatments and heritability estimates of test environments in Nigeria, 2015 to 2017.

S/N	Location	Year	Treatment	Heritability (%)
1	Bagauda	2017	Optimal conditions	30
2	Ikenne	2015	Drought	60
3	Ikenne	2016	Drought	61
4	Ikenne	2017	Drought	55
5	Ikenne	2016	Optimal conditions	69
6	Ikenne	2017	Optimal conditions	58
7	Mokwa	2016	Optimal conditions	60
8	Mokwa	2016	*Striga* infestation	58
9	Mokwa	2017	*Striga* infestation	56
